# Severe axonal neuropathy is a late manifestation of SPG11

**DOI:** 10.1007/s00415-016-8254-5

**Published:** 2016-08-20

**Authors:** Andreea Manole, Viorica Chelban, Nourelhoda A. Haridy, Sherifa A. Hamed, Andrés Berardo, Mary M. Reilly, Henry Houlden

**Affiliations:** 1 Department of Molecular Neuroscience and Neurogenetics Laboratory, UCL Institute of Neurology, Queen Square, London, WC1N 3BG UK; 2 MRC Centre for Neuromuscular Diseases, UCL Institute of Neurology, Queen Square, London, WC1N 3BG UK; 3 Department of Neurology, Medical University N. Testemitanu, Chisinau, Republic of Moldova; 4 Instituto de Neurociencias Conci Carpinella, Laboratorio de Neurobiologìa, Instituto de Investigaciónes Medicas “Mercedes y Martín Ferreyra”, INIMEC-CONICET-UNC, Córdoba, Argentina; 5 Department of Neurology and Psychiatry, Faculty of Medicine, Assiut University Hospital, Assiut, Egypt

**Keywords:** Axonal neuropathy, Spatacsin, CMT2, Gene, Genetics, SPG11

## Abstract

Complex hereditary spastic paraplegia (HSP) is a clinically heterogeneous group of disorders usually inherited in an autosomal recessive manner. In the past, complex recessive spastic paraplegias have been frequently associated with SPG11 mutations but also with defects in SPG15, SPG7 and a handful of other rare genes. Pleiotropy exists in HSP genes, exemplified in the recent association of SPG11 mutations with CMT2. In this study, we performed whole exome sequence analysis and identified two siblings with novel compound heterozygous frameshift SPG11 mutations. The mutations segregated with disease were not present in control databases and analysis of skin fibroblast derived mRNA indicated that the SPG11 truncated mRNA species were not degraded significantly by non-sense mediated mRNA decay. These siblings had severe early-onset spastic paraplegia but later in their disease developed severe axonal neuropathy, neuropathic pain and blue/black foot discolouration likely caused by a combination of the severe neuropathy with autonomic dysfunction and peripheral oedema. We also identified a similar late-onset axonal neuropathy in a Cypriot SPG11 family. Although neuropathy is occasionally present in SPG11, in our SPG11 patients reported here it was particularly severe, highlighting the association of axonal neuropathy with SPG11 and the late manifestation of axonal peripheral nerve damage.

**Electronic supplementary material:**

The online version of this article (doi:10.1007/s00415-016-8254-5) contains supplementary material, which is available to authorized users.

## Introduction

The hereditary spastic paraplegias (HSPs) are heterogeneous inherited neurological disorders, resulting in progressive spasticity of the limbs, bladder dysfunction and walking difficulties. Other features such as weakness, ataxia, cognitive decline and peripheral neuropathy are present in complex HSP. They are classified genetically as autosomal dominant, autosomal recessive and X-linked HSP; and clinically as pure or complicated HSP, based upon the absence (pure) or presence (complex) of additional clinical features. To date, more than 70 different disease-loci and more than 50 spastic paraplegia genes (SPGs) have been identified [1, 2]. Autosomal recessive HSP with a thin corpus callosum is a common subtype of complex HSP. Mutations in *SPG11* (encoding for Spastacsin), comprise about 70 % of these cases [2–4] and the onset is usually in the teenage years [5–12].

In the past, axonal neuropathy has been infrequently associated with complex HSP and SPG11 mutations, although this is usually mild [4, 13]. Recently mutations in SPG11 have been shown to cause Charcot–Marie–Tooth disease type 2 (CMT2) as a distinct clinical phenotype [14]. Spatacsin is expressed ubiquitously in the nervous system [1] and the sural nerve biopsy pathology in typical SPG11 cases showed a loss of unmyelinated nerve fibers and accumulation of intra-axonal pleomorphic membranous material. In CMT2 cases sural nerve biopsies showed similar intra-axonal inclusions but also predominantly loss of large myelinated fibers, mainly in fibers of large caliber, in line with the diagnosis of CMT2 [14]. Although the role of the protein is unknown, it seems to be important to the survival of neurons [15].

In this paper we describe novel frameshift mutations in SPG11, identified by whole exome sequencing, segregating with the disease and investigated in mRNA. The causative mutations were found in two sisters with complex HSP consistent with SPG11, associated with the late manifestation of severe axonal neuropathy. A similar presentation was found in a second, unrelated Cypriot family.

## Methods

In family F1, two sisters with complex HSP, with clinical features as described below were investigated. Informed consent (UCLH: N99/103) was obtained from all individuals and the institutional review boards at the participating medical centers approved the study. Acquired spastic paraplegia was excluded and neurophysiological studies, MRI scans and skin biopsies were performed using standard methods. Genomic DNA samples from the two affected individuals and two unaffected relatives were used for molecular genetic analyses (supplementary material). The mutation in the Cypriot family was previously reported [4].

Exome sequencing was performed as previously described [16–18] using the Agilent SureSelect kit and run on the Illumina HiSeq2500. Sequences were aligned with the Burrows-Wheeler Aligner, duplicates were removed with Picard, indels aligned and base quality scores recalibrated with the Genome Analysis Toolkit (GATK). The average sequencing depth was 55-fold with variants being filtered according to pathogenicity, inheritance pattern, and segregation in the family. We confirmed causative variant candidates by the Sanger sequencing method using an automatic genetic analyzer. SPG11 PCR primers were designed using Primer3 so that the PCR products would span whole exons and about 35 bp of flanking introns (http://primer3.ut.ee/). Primer sequences are listed in the supplementary file. Touchdown PCR was done using the PCR Master Mix (Roche) and is described in more detail in the supplementary table. PCR amplification products were cleaned with ExoAp. The purified PCR products was split into two and sequenced bidirectionally with the original primers that were used to amplify the region of interest and Big Dye Terminator Kit v.3.1 (Applied Biosystems) [19, 20]. Conditions were as follows: 25 cycles of denaturation at 95 °C for 10 s, annealing at 50 °C for 5 s and extension at 60 °C for 4 min. Sequencing reactions were cleaned using CleanSEQ SPRI beads according to the manufacturer’s protocol (Agencourt). Sequencing was performed using a 3730 DNA Analyzer (Applied Biosystems). SPG11 mutation positions are based on NCBI reference sequences: NM_001160227, NP_001153699 (www.ncbi.nlm.nih.gov).

### RNA isolation and reverse transcriptase

Total RNA was extracted from cultured skin fibroblasts using Direct-zol™ RNA Miniprep (Zymo Research, USA) according to the manufacturer’s instructions and reverse transcribed to first strand cDNA by using random primers and Moloney murine leukemia virus (M-MLV) reverse transcriptase (Promega Corporation, Madison, WI, USA). The concentration and purity of RNA was determined spectrophotometrically. PCR amplification and sequencing of cDNA: three primer pairs were designed in flanking exons of those of interest to amplify the coding sequence of exons 22 and 30 of SPG11 mRNA (Supplementary material). Sanger sequencing was performed as above.

## Results

### Clinical features

**Fig. 1:**
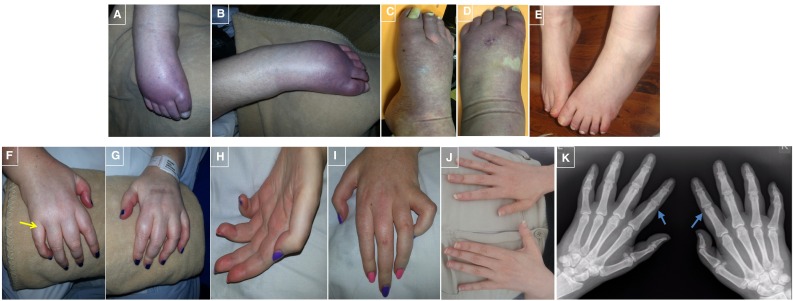
Photographs of the feet in SPG11 patients F1II:1 and F1II:2 (A and B) and F1AII:2 (C and D) showing peripheral oedema and marked discolouration of the feet that reverses with massage. Figure E are feet from a typical SPG11 patient in her mid 30s (Family 4, Tables 1, 2). Figure F to I shows photographs of the hands in the proband F1II:1 and F1II:2 showing generalized tapering of the fingers and swelling of the proximal phalanx, red discolouration of the hands, more marked in the palms. Figure J are hands from a typical SPG11 patient, Family 4. Figure K shows an x-ray of the hands (patient F1II:1) with mild narrowing of the proximal interphalangeal joints bilaterally (arrows) compatible with mild osteoarthritis

The proband and her sister from family F1 (II:1 and II:2; Fig. 1) were the first and second children of healthy, non-consanguineous parents of British descent. At ages 19 and 16 years, respectively, they presented with progressive walking difficulties with additional features of bulbar dysarthria, cognitive problems, limb weakness (proximal power MRC 2/5 and distal 1/5 on examination at the age of 39 and 36, respectively), bladder dysfunction and optic atrophy. At first in their early 20s, limb tone was increased and reflexes were brisk with extensor plantars and no sensory abnormalities but at the ages 39 and 36 reflexes were absent and there was distal sensory loss to pin prick, light touch and temperature. Their fingers were long and tapering, palms were discolored and there was significant limb pain. In addition to having leg oedema the feet of both sisters had strikingly black coloration which improved with massage (Fig. 1). Cognitive tests showed IQs of 54 for the proband, and although only a very limited assessment was possible in the sister due to severe motor and language impairment in the context of complex HSP. The overall picture was one of the global and severe cognitive impairments, occurring at an early age with relative sparing of visual perceptual functions and normal hearing. Pulses were difficult to palpate with the peripheral oedema but capillary refill was normal as was blood pressure testing.

**Fig. 2:**
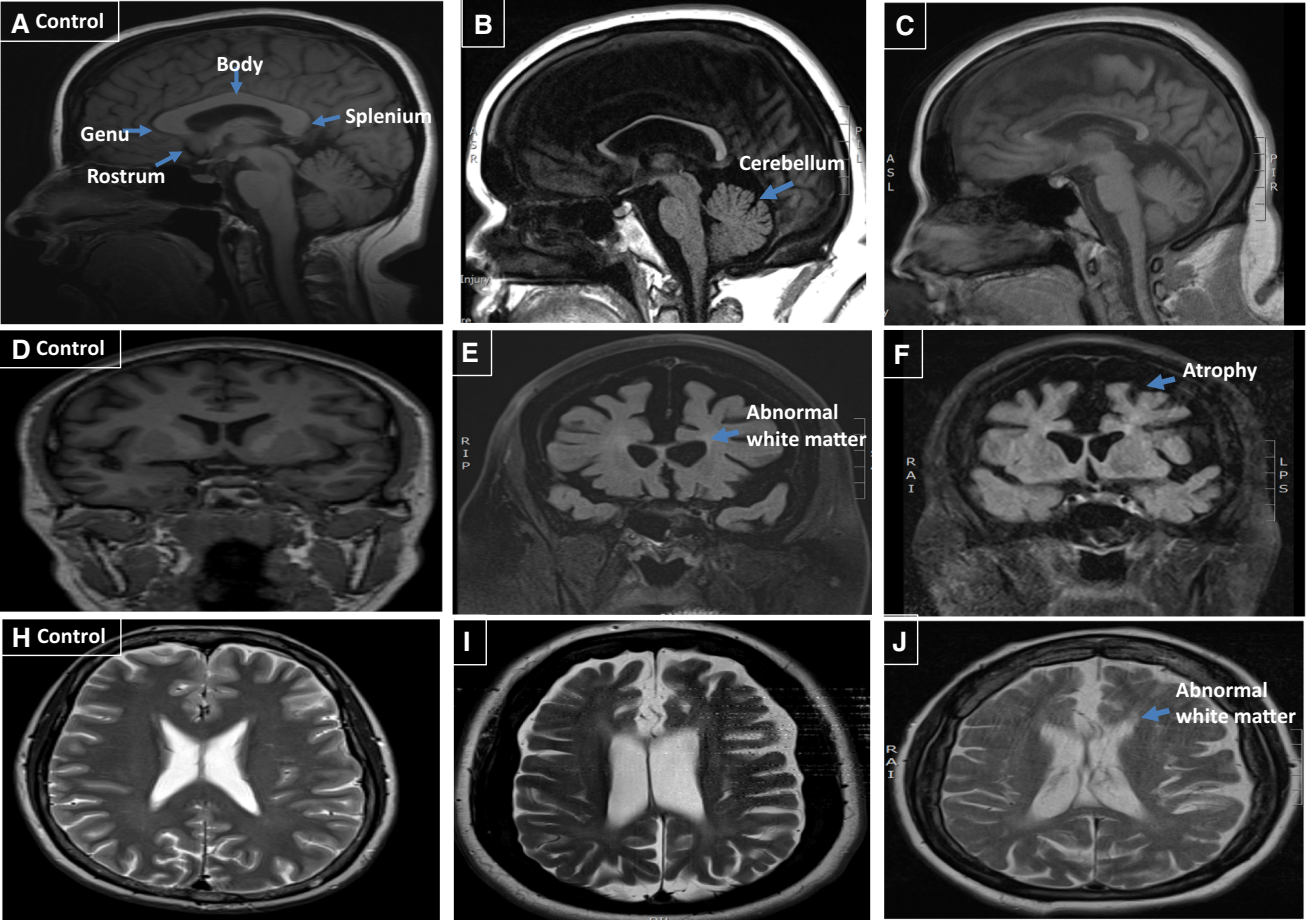
Sagittal MRI sequence; **a** control MRI with corpus callosum labeling. **b** Proband II:1 and **c** case II:2 showing thinning of the corpus callosum and cerebral atrophy. Coronal MRI sequence; **d** control. **e** Proband II:1 and **f** II:2 showing generalized atrophy with periventicular white matter abnormalities. Axial MRI sequence; **h** control. **i** Proband II:1 and **j** II:2 showing periventicular white matter abnormalities

The proband in the Cypriot SPG11 (F1AII:2) family had similar clinical features and initially presented at the age of 21 years with a complex HSP phenotype. When this patient was re-examined and investigated at the age of 42 years she had low tone and absent reflexes along with likely distal sensory abnormalities that were difficult to define with the severe cognitive features but these occurred early in the disease. Her feet showed similar discolouration but she did not suffer from any pain (Figs. 1, 2; Tables 1, 2) and strikingly different to typical SPG11 patients.

**Table 1:** SPG11 variants identified with clinical details

Proband number	Variant	Variant type	Ethnic origin	Consanguinity	Family history	Age at onset	Current age	Gender
F1	c.3729delC, p.S1243 fs/	Compound heterozygous#	UK	No	Yes	16	39	F
	c.5148dupA, p.H1717 fs							
F1A	c.6658_6659delAT, p.M2220Dfs*27	Homozygous#	Cypriot	No	Yes	21	42	F
F2	c.2146C>T, p.Q716*	Homozygous	Pakistan	Yes	No	Child	26	F
F4	c.7000G>C, p.A2334P/c.3146-1G>C	Compound heterozygous#	Italian/Argentina	No	Yes	23	39	F
F5	c.3809T>A, p.V1270D	Homozygous#	Turkish	Yes	Yes	12	18	M
F6	c.5769delT, p.S1923Rfs*28	Homozygous#	Kenya/India/UK	Yes	Yes	20	33	F
F8	c.3623C>T, p.P1208L/c.852_856delCTTAA, p.N284Kfs*14	Compound heterozygous#	UK	No	No	19	25	F
F16	c.398delG, p.C133Lfs*22	Homozygous#	Iranian	Yes	No	17	35	F
F26	c.5399_5407delAGATinsTGGAGGAG, p.Q1800Lfs*31	Homozygous	Pakistan	Yes	Yes	13	33	F

SPG11 variants were labeled according to the transcript NM_025137.3 using the standard mutation nomenclature used in molecular diagnostics. See main text for discussion on pathogenicity. Families 2, 4, 5, 6, 8, 16, and 26 [4] are added for comparison to families F1 and F1A reported here. * Nonsense, *del* deletion, *N/A* not available, # other family members available for segregation

**Table 2:** Nerve conduction studies (NCS) and electromyography (EMG) in the proband from family 1 (II:1) and family 1A (II:2) reported here as compared with other probands (F2, F4-6, F8, F16 and F26) from different SPG11 families (Table 1)

Case	Motor nerve conduction studies									Sensory nerve conduction studies						EMG		Summary findings of NCS and EMG
	Median nerve			Ulnar nerve			Tibial nerve			Median nerve		Ulnar nerve		Sural nerve		Limbs. Upper: lower		
	DML (msec)	CMAP amp (uV)	MCV (m/s)	Distal lat (msec)	CMAP amp (uV)	MCV (m/s)	Distal lat (msec)	CMAP amp (uV)	MCV (m/s)	SNAP amp (uV)	SCV (m/s)	SNAP amp (uV)	SCV (m/s)	SNAP amp (uV)	SCV (m/s)	Chronic denervation		
SPG 11 patients																		
F1 II:1	3.3	2.7	48	2.8	2.4	63	5.1	0.3	N/A	12	68	6	60	NR	NR	Yes	Yes	Severe sensory-motor axonal neuropathy
F1A II:2	4.4	0.4	41	N/A	NR	N/A	4.7	0.1	36	17	50	6	51	NR	NR	Yes	Yes	Severe sensory-motor axonal neuropathy
F2	3.3	10.7	55	2.4	11.9	58	4	3.9	53	25	62.5	23	59	14	41.5	No	No	Normal NCS/EMG
F4	3.7	0.9	40	4.1	3.7	42	5.5	3.6	43	N/A	N/A	N/A	N/A	11.6	35	Mod	Mod	Mild axonal neuropathy
F5	4	4.9	51	2.4	10	61	3.4	5.2	48	28	61	13	67	11	55	No	No	Normal NCS/EMG
F6	3.2	8.3	56	2.3	8.9	62	2.8	3.7	45	23	65	15	64	12	48	N/A	No	Normal NCS/EMG
F8	3.9	7.3	64	N/A	N/A	N/A	4.8	5.3	45	19	61	9	50	6	55	No	Mild	Mild axonal neuropathy
F16	4.1	6.6	52	N/A	N/A	N/A	5	3.4	42.5	15	57.5	14	53.5	14	41.5	No	No	Normal NCS/EMG
F26	3.6	6.1	53	3.8	3.8	43	5.9	3.3	35	28	54	19	56	9	40	Mod	Mod	Mild axonal neuropathy

*NR* no response, *Amp* amplitude, *lat* latency, *DML* distal motor latency, *CMAP* compound muscle action potential, *mod* moderate, *μV* microvolts, *m/s* meters per second, *msec* milliseconds

**Fig. 3:**
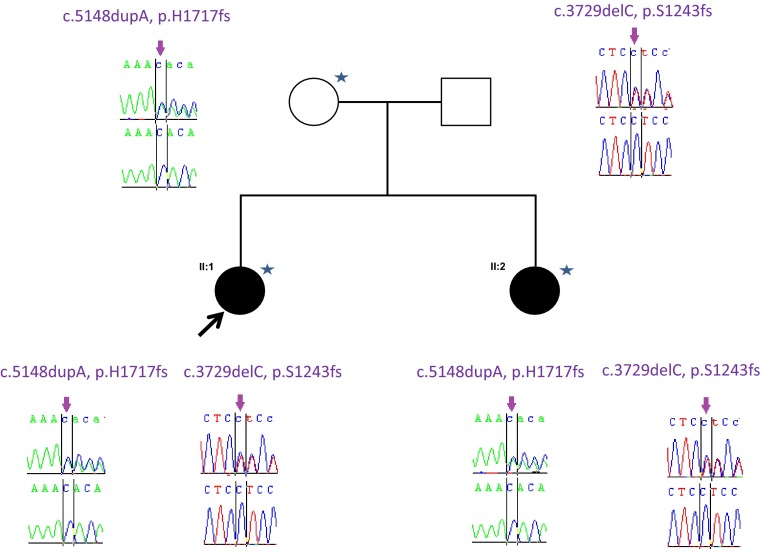
Pedigree of the F1 *SPG11* family. Open symbols represent unaffected individuals and filled symbols represent affected individuals. The proband is indicated by an arrow. Stars indicate individuals whose DNA was used for whole exome sequencing and whose skin biopsies were used for fibroblast studies. Sanger sequencing chromatograms surround the symbols and show segregation of mutations. Black bar encompass the sites of interest (mutation over control site)

Brain MRIs in both cases showed that the corpus callosum was thin with periventricular white matter changes and possible cerebellum atrophy (Fig. 3). Nerve conduction studies show a severe length-dependent axonal sensory-motor polyneuropathy affecting both lower and upper limbs. The neuropathy was compared with other typical SPG11 cases with complex HSP and was significantly worse (Table 2).

### Genetic studies

**Fig. 4:**
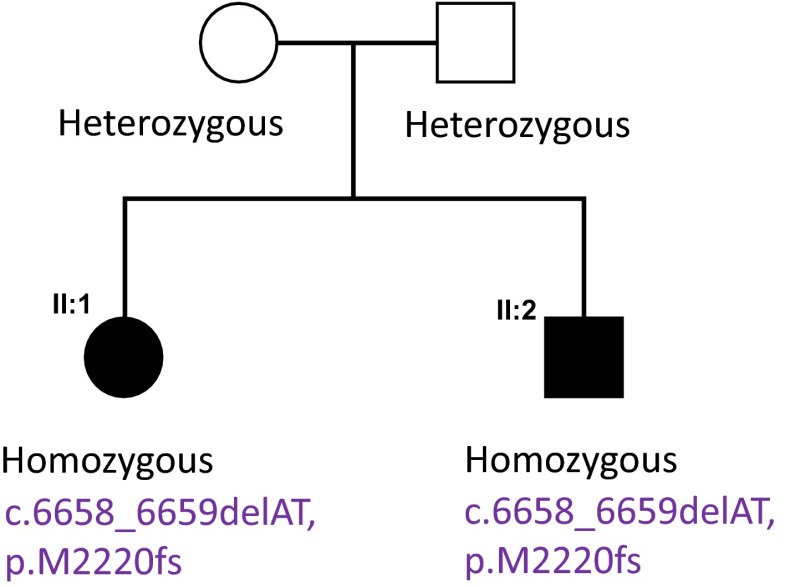
Pedigree of F1A Cypriot SPG11 family. Open symbols represent unaffected individuals and filled symbols represent affected individuals. SPG11 mutation has been previously reported

**Fig. 5:**
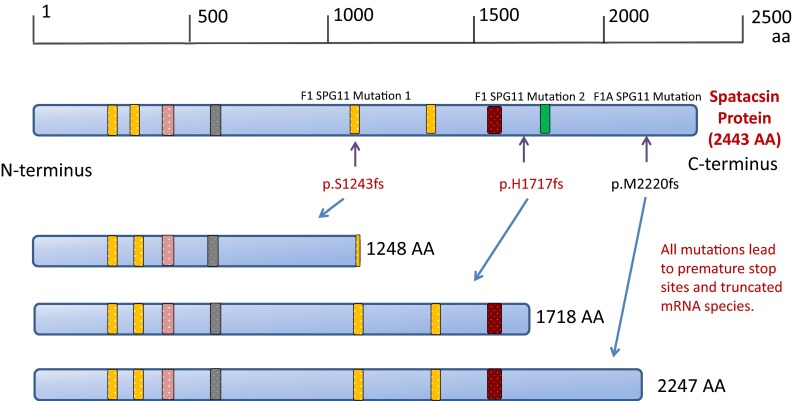
Schematic representation of the SPG11 protein and position of known (black) and novel (red) mutations. Mutation 1 is predicted to truncate the full length protein from 2443 amino acids (AA) to 1248 AA, mutation 2 truncates to 1718 AA and mutation 3 to 2247 AA. Putative functional domains are depicted as rectangles, and their positions within the amino acid sequence are indicated: the transmembrane domain (yellow box; positions, 163–194, 200–240, 1239–1267, and 1471–1493), glycosyl hydroxylase F1 signature (pink box; position, 482–490), leucine zipper (gray box; position, 611–632), coil–coil domain (brown box; position, 1556–1590), and Myb domain (green box; position 1766–1774). Arrows indicate truncating mutations; dotted arrows indicate missense mutations. Aa = amino acids

**Fig. 6:**
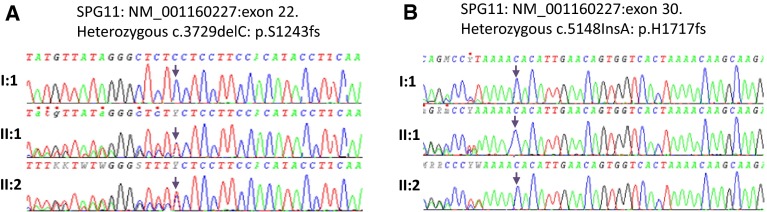
A. cDNA sequencing chromatograms of the heterozygous exon 22 SPG11 mutation in the mother (I:1 same as control sequence) and two affected siblings (II:1 and II:2) with deletion of a C (arrows). B. cDNA sequencing chromatograms of the heterozygous exon 30 SPG11 mutation in the mother (I:1 same as affected sequence) and two affected siblings (II:1 and II:2) with the insertion of an A (arrow). The mutation is clearly visible in both chromatograms and the peak heights are the same as genomic DNA (Fig. 3)

To identify the underlying genetic cause, we applied whole-exome sequencing (WES) on the proband (II:1), her affected sister (II:2) and her mother (I:1). Analysis focused on nonsynonymous, splice-site and coding indel variants with a minor allele frequency (MAF) of <0.5 % in the Exome Aggregation Consortium (EXaC; www.exac.broadinstitute.org), Exome Variant Server (EVS; http://evs.gs.washington.edu) and 1000 Genomes databases (1000G; http://www.1000genomes.org). From the variants that met these filtering criteria in the proband, a few co-segregated under an autosomal recessive model and at least one was present in the mother. Of these variants, only two involved a gene associated with complex HSP, i.e., the *SPG11* gene. These variants were validated by Sanger sequencing. Segregation analysis confirmed that both affected siblings and their unaffected father were heterozygotes for the *SPG11* variant in exon 22, c.3729delC. Moreover, both affected sisters and their mother were heterozygotes for the variant in exon 30 c.5148dupA, also indicating that these two variants were located on the different alleles (Fig. 4). The variant in exon 22 is a one bp deletion which creates a frameshift disrupting the sequence from codon S1243. The new reading frame ends in a stop 6 codon positions downstream. Similarly the variant in exon 30 is a one bp duplication which creates a frameshift disrupting the sequence from codon H1717. The new reading frame ends in a stop 3 position downstream. To analyze the consequence of these mutations, we isolated RNA from the fibroblasts of the two affected sisters and their mother, extracted RNA and carried out RT-PCR. From our data the mutant allele is clearly present in the cDNA (Figs. 5, 6) and likely to exist as a truncated protein, indicating that very little RNA has been targeted for non-sense mediated mRNA decay (Fig. 6). The Cypriot family were found to have a homozygous c.6658_6659delAT, p.M2220Dfs*27 mutation. This is consistent with a typical SPG11 loss of function mutation and the family originated from a small village in Cyprus.

## Discussion

In this study, we identified novel compound heterozygous mutations in SPG11 in a complex HSP family with thin corpus callosum and severe axonal sensory-motor polyneuropathy as a late manifestation of the disease. Moreover they had feet which were oedematous and strikingly blue–black in color as a likely result of fluid dependency and peripheral neuropathy. A recent study by Montecchiani et al. [14] showed that SPG11 is the causative gene of a wide spectrum of clinical features, including autosomal recessive CMT2. The severe neuropathy in our case is consistent with neuropathy being a major feature in some, but not all patients with SPG11 mutations and a complex HSP phenotype as is confirmed in the Cypriot family. An interesting aspect of the two SPG11 families discussed here is the initial clinical presentation as complex HSP with a later development of an axonal neuropathy clinically and electrophysiologically. As in SPG11 associated with complex HSP, the variants described by Montecchiani et al. [14] were scattered throughout the entire DNA sequence, without evidence of ‘hot spots’, and 93 % were truncating mutations. Our findings are consistent with this and other previous studies describing mostly mutations leading to the truncation of the SPG11 protein and consequent loss of function mechanism [21].

In Family 1 based on our cDNA sequencing data, we would expect the protein to be truncated and only little of the mRNA to be targeted for non-sense mediated decay. In our case, one of the truncated forms of the protein is missing the Myb domain, and the other one is missing both the Myb and the coil–coil domains whose presence may suggest a role of Spatacsin in regulation of gene expression [22]. In terms of pathology, sural nerve biopsy of SPG11 has previously shown loss of unmyelinated nerve fibers and accumulation of intra-axonal pleomorphic membranous material [23]. Also, axonal trafficking of vesicles was demonstrated to be impaired in neurons derived from induced pluripotent stem cells of SPG11 patients [24]. Another alteration is in the secretory pathway. The SPG11 protein Spatacsin has been shown to account for proteins involved in the formation of lysosomes and moreover to interact with components of the AP5 complex involved in membrane sorting of late endosomes [25, 26].

As our understanding of the hereditary spastic paraplegias increases it is clear that the clinical features are very heterogeneous and the spectrum of signs in disease genes such as SPG11 can vary substantially. It is noteworthy for clinicians to consider SPG11 testing in early onset complex HSP or where there is a combination of severe neuropathy and spasticity.

In conclusion, we provide support for the use of whole exome sequencing as a diagnostic tool for identification of mutations in conditions with complex and wide presentations such as HSP. Furthermore, we extend the findings that mutations in SPG11 are the cause of a spectrum of clinical features including the late manifestation of severe axonal neuropathy.

## Electronic supplementary material

Below is the link to the electronic supplementary material.

Supplementary material 1 (DOCX 52 kb)
