# Supplementary material for: Severe axonal neuropathy is a late manifestation of SPG11
Source: J Neurol. 2016 Aug 20;263(11):2278–86. doi: 10.1007/s00415-016-8254-5 (PMC5065903; doi:10.1007/s00415-016-8254-5)
Supplement: Supplementary file 1 — Supplementary material 1 (DOCX 52 kb) [file 415_2016_8254_MOESM1_ESM.docx]

**Supplemental tables and information**

| Table S1, *SPG11* PCR primer sequences used for PCR and sequencing | | | |
| --- | --- | --- | --- |
| 1F | AGTTAAGCAGAGTGGGAC | 21F | TCATTAGAGAGTTCCATGTG |
| 1R | TGAATAATGAAAGAATCAGC | 21R | AAGTGATCCTCCTGCTTC |
| 2F | ACTTATTCAACCCTTCTACC | 22F | CTGATTTAATTCCATTTGC |
| 2R | ACCTCTATGACTTTTCCTG | 22R | AAACACTCACAATTCAATG |
| 3F | ATCAAATCAAATGTACCAG | 23F | CTATTCAAACAGAAATGCTC |
| 3R | TTCCTATATCCCAGCTCC | 23R | ACACCAGAGTTGTTCAAAG |
| 4F | ACTTCATAGTCATCTGGTTG | 24F | TTTCCAGTTCAAGCTAAAG |
| 4R | TAGCCAGTGGTCAGTTATAC | 24R | AGGCCTATGTCATGTCTAC |
| 5F | TTAACAGGAGCAGTAGTAAC | 25F | CTCTAACATTTTCAGCTCC |
| 5R | TTTAATGAAAGGGTACAGC | 25R | ATTATCAGCCTTTCCTGTC |
| 6F | TTTTAAGAACATCTTTGCC | 26F | CAGGAAATATCCATCTGTAG |
| 6R | GAGGCAGAAGTAAAATACAG | 26R | CATCATTATCTGTTGTTGG |
| 7F | AAAACCATGATGAGTTAAG | 27F | TATTTGGCATTATTCATTG |
| 7R | TAAGCAGAGTTAGGGTAATG | 27R | CGAGTGAGACACCAAGTC |
| 8F | AGTCTCACCACATTAAATTC | 28F | TTTCTTAGAGGTTCCCAG |
| 8R | ATGACTAAGTTTTGGCAAG | 28R | AATTTCCTAACTACCCCTC |
| 9F | AATAAGACATTGGTCCTTG | 29F | TTTTCCTTTAACCACATTAC |
| 9R | TACCCAAATGTAGTAAATGG | 29R | CAGATCAAGACCATCCTG |
| 10F | TACCAAGTTTGAATTTTGAC | 30F | GATATACTTAGGGAACATTAGC |
| 10R | GAATTCTGTTTCTTTCTATTG | 30-1F | AACTTGGAGAAGGAAAAC |
| 11F | AAATGTATAATCCCATGTTG | 30-1R | GAGCCATAGAGAGCTAGG |
| 11R | AATGTCATTATTTCTTAGTGTC | 30-2F | GAAATCAGGAGGAAACAG |
| 12F | AGGGTGTTTCTGTATCTAAC | 30-2R | CTTCCTTCTTGGAGAXAG |
| 12R | TCTTCCAAGGTTTTCTTC | 31F | ATTTCCTGGAAGAGGGCAAT |
| 13F | AATAATCTTTAATTCCCAGC | 31R | TTATCATCTAAAAGGCTGAC |
| 13R | AGTTCCACATAAGAAACTTG | 32F | CTGCTGAGGGTGAGGAGTCT |
| 14F | AAAAGAGTGGATGTTCTTG | 32R | TGCAATCCAGAAACTTGAGAGA |
| 14R | AATATTATTTCCCGAAAGG | 33F | AAGGGTTTCAAGCTCAGCAA |
| 15F | CACAGCGAGATCCTGTCTCA | 33R | TAGGCATCCAGAGCAGGAAC |
| 15R | GGATTTATGGCATTTCAAAGGA | 34F | CTTTGAGGATTGTGCCATGA |
| 16F | TTTTAAGTAATTGAGACCTAAAC | 34R | GCCCAGCCAACTCTCAAGTA |
| 16R | AAAGTCACATTCAGGAGTC | 35F | GGCATCTGAAAGCAACCACT |
| 17F | TTAAATGCTAATCATCGC | 35R | CTTGGGGAGGTCCCTAATTC |
| 17R | TTCACAAGTTTAATACCATTC | 36F | TTTAAGGTTTCTCCCTTTAG |
| 18F | TCTCTCAGTTCATTGTTACC | 36R | ACATAGTCAAACCCCATC |
| 18R | TTTAAATTCAGCCTTATCC | 37-38F | CTATTCTAGCCACGATCAC |
| 19F | ATCTTGTTTCACAAGGTTC | 37-38R | GACCTTACCTCTGGGTTC |
| 19R | GAAAGATCTAGAGTGATTTCTG | 39F | TTGTTTTCTACAACAGTTTG |
| 20F | GAGCAGACTACCTCTGAAG | 39R | TTAGCCATAAAATTCTTACAC |
| 20R | GAAAACTAGATTGGCATTAC | 40F | TGGGCAACAGAGTAAGAC |
| 21F | TCATTAGAGAGTTCCATGTG | 40R | GCTGTCCTGAGGAAGAGGAA |
| 21R | AAGTGATCCTCCTGCTTC |  |  |
| 22F | CTGATTTAATTCCATTTGC |  |  |
| 22R | AAACACTCACAATTCAATG |  |  |
| 23F | CTATTCAAACAGAAATGCTC |  |  |
| 23R | ACACCAGAGTTGTTCAAAG |  |  |

| Table S2, *SPG11* PCR program | | |
| --- | --- | --- |
| Temperature (^o^C) | **Time (min)** | **Number of cycles** |
| 94 | 01:00 |  |
| 94 | 00:30 | x15 |
| 58 | 00:30 |  |
| 72 | 00:30 |  |
| 94 | 00:30 | x16 |
| 58* | 00:30 |  |
| 72 | 00:30 |  |
| 94 | 00:30 | x14 |
| 52 | 00:30 |  |
| 72 | 00:30 |  |
| 72 | 05:00 |  |

*Reducing temperature in each cycle.

| Table S1, *SPG11* PCR primer sequences used for PCR and sequencing of cDNA | | | |
| --- | --- | --- | --- |
| 21F | AGCTATAGGAGATGCATGGAGT | **23R** | TGGCTGCTAGATTCACTGGA |
| 29F | AGCTGAGTTACCTGTGGACA | **30_1R** | CACCACTAGTTGAGATCTGTCG |
| 30_1F | ACGTGGTGCCCTTGGATAAG | **31R** | CTGCTTATGTCTGCTGTGGAC |

| Table S2, *SPG11* PCR program for cDNA | | |
| --- | --- | --- |
| Temperature (^o^C) | **Time (min)** | **Number of cycles** |
| 94 | 01:00 |  |
| 94 | 00:30 | x15 |
| 70 | 00:30 |  |
| 72 | 00:30 |  |
| 94 | 00:30 | x16 |
| 70* | 00:30 |  |
| 72 | 00:30 |  |
| 94 | 00:30 | x14 |
| 65 | 00:30 |  |
| 72 | 00:30 |  |
| 72 | 05:00 |  |

*Reducing temperature in each cycle.
